# The Physicians’ Mutual Benefit Association of Texas

**Published:** 1886-03

**Authors:** 


### THE P. M. B. A. OF TEXAS.

DURING the session of the Texas State Medical Association, which will be held at Dallas, in April 24th prox., a meeting will be called of all the members of the Physicians' Mutual Benefit Association who are present, during a recess of the former. The Secretary and Treasurer will make a report of the business of the Society and its present status. On that occasion, too, it is proposed to elect a Board of Directors or Trustees; and, perhaps, to make some alterations of, or additions to the Constitution and By-Laws. Members will please make a note of this meeting.

